# Immune (Cell) Derived Exosome Mimetics (IDEM) as a Treatment for Ovarian Cancer

**DOI:** 10.3389/fcell.2020.553576

**Published:** 2020-09-17

**Authors:** Simone Pisano, Irene Pierini, Jianhua Gu, Andrea Gazze, Lewis Webb Francis, Deyarina Gonzalez, Robert Steven Conlan, Bruna Corradetti

**Affiliations:** ^1^Department of Nanomedicine, Houston Methodist Research Institute, Houston, TX, United States; ^2^Centre for NanoHealth, Swansea University Medical School, Swansea, United Kingdom

**Keywords:** exosomes, ovarian cancer, mimetics, immune system, synthetic

## Abstract

Exosomes are physiologically secreted nanoparticles recently established as natural delivery systems involved in cell-to-cell communication and content exchange. Due to their inherent targeting potential, exosomes are currently being harnessed for the development of anti-cancer therapeutics. Clinical trials evaluating their effectiveness are demonstrating safety and promising outcomes. However, challenging large-scale production, isolation, modification and purification of exosomes are current limitations for the use of naturally occurring exosomes in the clinic. Exosome mimetics hold the promise to improve the delivery of bioactive molecules with therapeutic efficacy, while achieving scalability and increasing bioavailability. In this study, we propose the development of Immune Derived Exosome Mimetics (IDEM) as a scalable approach to target and defeat ovarian cancer cells. IDEM were fabricated from monocytic cells by combining sequential filtration steps through filter membranes with different porosity and size exclusion chromatography columns. The physiochemical and molecular characteristics of IDEM were compared to those of natural exosomes (EXO). Nanoparticle Tracking Analysis confirmed a 2.48-fold increase in the IDEM production yields compared to EXO, with similar exosomal markers profiles (CD81, CD63) as demonstrated by flow cytometry and ELISA. To exploit the prospective of IDEM to deliver chemotherapeutics, doxorubicin (DOXO) was used as a model drug. IDEM showed higher encapsulation efficiency and drug release over time compared to EXO. The uptake of both formulations by SKOV-3 ovarian cancer cells was assessed by confocal microscopy and flow cytometry, showing an incremental drug uptake over time. The analysis of the cytotoxic and apoptotic effect of DOXO-loaded nanoparticles both in 2D and 3D culture systems proved IDEM as a more efficient system as compared to free DOXO, unraveling the advantage of IDEM in reducing side-effects while increasing cytotoxicity of targeted cells, by delivering smaller amount of the chemotherapeutic agent. The high yields of IDEM obtained compared to natural exosomes together with the time-effectiveness and reproducibility of their production method make this approach potentially exploitable for clinical applications. Most importantly, the appreciable cytotoxic effect observed on ovarian cancer *in vitro* systems sets the ground for the development of compelling nanotherapeutic candidates for the treatment of this malady and will be further evaluated.

## Introduction

Exosomes (EXO) are well established throughout the scientific community as paramount mediators of cell-to-cell communication and content exchange (Tkach and Théry, 2016). They mediate physiological processes but have also been associated with many different pathologies, due to their capacities of transporting different types of cargos (mainly RNAs, DNA, lipids and proteins). The molecular composition and function of exosomes resemble those of their parental cells, with inherent targeting mechanisms and a lipophilic core that compartmentalizes native materials (Record et al., 2014) and provides structural stability to the cargo (De Toro et al., 2015; Isola and Chen, 2016; Keerthikumar et al., 2016).

Currently, a lot of effort is being put into the development of approaches capable of effectively harnessing exosomes properties (i.e., size, molecular content, low immunogenicity) for their clinical application in the treatment or diagnosis of a plethora of diseases, including cancer. So far, exosomes have been used as diagnostic tools due to their molecular content (Tanaka et al., 2013; Wang et al., 2014) or as therapeutic options, as in the case of clinical grade exosomes produced to tackle pancreatic cancer (Mendt et al., 2018) or melanoma in the form of vaccines (Escudier et al., 2005). The exploitation of exosomes with different origin as reconfigurable delivery vehicles also offers opportunities compared to other nanoscopic drug delivery systems such as liposomes and polymeric nanoparticles (Conlan et al., 2017). Exosomes are non-immunogenic in nature due to similar composition as the body’s own cells and can be loaded with therapeutic moieties, as showed for some cancer applications. For example, macrophage-derived exosomes were loaded with the chemotherapeutic agent doxorubicin (DOXO) and showed the highest cytotoxicity in a pancreatic cancer model when compared to DOXO-loaded pancreatic cancer cell- and pancreatic stellate cell-derived exosomes, suggesting that donor cell-specific differences could influence their utility as drug delivery vectors (Kanchanapally et al., 2019).

However, regardless of their well-established characteristics and potential, the use of exosomes for clinical application is still encountering limitations due to some intrinsic drawbacks in their production and scalability. The low extraction yields, as well as the inadequate loading and encapsulation efficiencies represent some issues for the clinical scalability of this platform, including their use as drug delivery systems (Ha et al., 2016; Akuma et al., 2019). Other disadvantages are related to the different methods used for their isolation, such as low recovery and purity, high reagent costs, lack of protocol standardization, morphology and phenotype changes across different techniques (Li et al., 2019). The current alternatives to exosomes involve some well-established liposome-based platforms, which already are or will be heading toward a clinical application, with pegylated liposomal doxorubicin being the first to be used as a standard of care in the treatment of cancers such as ovarian, breast and melanoma (Bobo et al., 2016).

Due to the aforementioned exosomes limitations, a drive toward a synthetic or semi-synthetic approach to exosomes has also gained momentum, and some researchers have demonstrated high yields of production and purity (Conlan et al., 2017). As an example, Goh et al. (2017a, b) proposed a biomimetic cell-derived platform obtained after shearing immune cells (U937 cell line) through sequential filtering, and obtained high yields of doxorubicin encapsulation that were tested on preclinical models of colon adenocarcinoma. Similar results were obtained using other cell types, such as macrophages (Raw264.7 cell line) and embryonic stem cells, to produce mimetic nanoparticles with a cytotoxic effect on malignant cancer models (Jang et al., 2013; Jo et al., 2014). In particular, Jang et al. demonstrated that membrane-derived nanovesicles are efficient for the targeted delivery of chemotherapeutics both *in vitro* and *in vivo*, regardless of whether they are produced naturally or artificially from cells (Jang et al., 2013).

Among the different tumors semi-synthetic and biomimetic platforms have been applied to, to the best of our knowledge no such approach has been developed or tested for the treatment of ovarian cancer. Ovarian cancer is the most lethal gynecological malignancy (Siegel et al., 2020) and responds poorly to the standard therapies and to immunotherapies (Ghisoni et al., 2019). Several efforts are currently ongoing to exploit the potential of nanomedicine, immunotherapy or even a combination of the two to better treat ovarian cancer patients (Corradetti et al., 2019). In particular, there is an urgent need for the development of therapeutic strategies able to exploit the physical and biological barriers present in the peritoneal cavity of metastatic ovarian cancer in order to precisely accumulate at the disease site (Nizzero et al., 2020). Due to their capacity to stimulate the immune system and to act as chemoattractants for other cell populations, exosomes derived from immune cells represent ideal candidates to boost an anti-cancer response in immunologically cold tumors such as ovarian cancer.

Here, we demonstrate the development of Immune Derived Exosome Mimetics (IDEM) as a scalable approach to target ovarian cancer cells. By subjecting cells to serial extrusion through filters with diminishing pore sizes (10 and 8 μm wide filter membranes) followed by purifications through size exclusion chromatography, we generated high quantities of IDEM retaining the distinctive physiochemical and molecular features of naturally occurring exosomes. As a proof of principle, the ThP-1 monocytic cell line was chosen to produce IDEM due to its high proliferation rate and wide applicability for the development of immunotherapy-based approaches (Corradetti, 2016). Furthermore, immune derived platforms could prove advantageous when applied to an *in vivo* system. A systematic comparison between the IDEM formulation with exosomes extracted from the medium of the same number of cells through well-established protocols was performed. To assess their potential use as delivery systems, IDEM were encapsulated with doxorubicin, as a model chemotherapeutic drug which is widely used in clinical settings for the treatment of ovarian cancer, and their therapeutic efficacy evaluated *in vitro* 2D and 3D ovarian cancer models.

## Materials and Methods

### Cell Culture

Monocytic cell line (ThP-1 cells) was purchased from ATCC. Cells were grown in Roswell Park Memorial Institute (RPMI)-1640 medium (ATCC 30-2001^TM^) containing 10% (v/v) fetal bovine serum, 100 U/mL penicillin, 0.1 mg/mL streptomycin. ThP-1 were maintained at a concentration of 1-5 × 10^5^ cells/ml for expansion. Ovarian cancer cells (SKOV-3 cells) were purchased from Sigma-Aldrich and cultured McCoy’s 5A media (Gibco) containing 10% (v/v) fetal bovine serum, 100 U/mL penicillin, 0.1 mg/mL streptomycin. Culture conditions were established at 37°C and 5% CO_2_.

### Exosome Production

#### Immune Derived Exosome Mimetics (IDEM) Synthesis

Immune derived exosome mimetics were synthesized by optimizing the procedure reported by Goh et al. (2017b), where cells are passed through porous membranes of decreasing size in order to be deconstructed and reconstructed consequentially. Briefly, ThP-1 cells (8.5 × 10^6^) were harvested and washed twice in PBS. The PBS-resuspended pellet was then filtered through 10 μm-filter Pierce^TM^ spin cups (ThermoFisher) and centrifuged at 14,000 × *g* for 10 min at 4°C. The pelleted flow-through was resuspended in PBS and the same process repeated. Consequently, the pellet was passed through 8 μm filters (Merck-Millipore) with the same centrifuge settings as before. The pellet was finally resuspended in 150 μL of 0.22 μm-filtered PBS and run through G-50 Sephadex high capacity spin columns (SigmaAldrich) for further purification of the solution. This solution was stored at −80°C or used for downstream applications.

#### Exosomes Extraction and Purification

ThP-1 cells (8.5 × 10^6^) were incubated overnight in 15ml RPMI-1640 media supplemented with Exo-Free FBS (FisherSci). Media was then collected and processed through a series of centrifugations to remove the cellular component (500 × *g* for 5 min) and any debris (2000 × *g* for 30 min). The remaining supernatant was passed through 0.22 μm PES membrane filter (CellTreat) and then concentrated using 10 KDa Amicon ultra centrifugal filters (Millipore). Total exosome isolation reagent (TEIR, Invitrogen) was then added in a 1:1 ratio to the volume obtained after the Amicon-based concentration process. The solution was mixed by vortexing for 30 s and incubated overnight at 4°C. The next day, the sample was centrifuge at 10,000 × *g* for 1 h at 4°C. The concentrated solution was centrifuged at 10,000 × *g* for 1 h at 4°C, and the pellet was resuspended in 0.22 μm filtered PBS. This solution was stored at −80°C or used for downstream applications.

### Exosomes and IDEM Characterization

#### Nanoparticles Tracking Analysis (NTA)

Exosome and IDEM samples were analyzed according to the MISEV2018 Minimal information for studies of extracellular vesicles (Théry et al., 2018). The NS300 Nanosight System (Malvern) was used to determine size and concentration. A 100X dilution in PBS was prepared for each sample. Briefly, five videos of 60 s each were recorded for each sample, and the threshold was kept constant at 5. All the experiments were performed using different batches of EXO/IDEM, and statistical analysis was based on at least 3 biological replicates. Size and concentration measurements were done using 11 different batches of EXO and IDEM.

#### Evaluation of Exosome-Associated Markers

CD63 quantitation was performed using the antibody-based ExoELISA-ULTRA Complete Kit (SystemBio) following the manufacturer’s indications. Briefly, EXO and IDEM were loaded on a 96-well plate provided by the company and the CD63 positive particle number was quantified against a pre-set exosomes standard curve. The number of CD63+ particles obtained was then compared to the total amount of particles (quantified by NTA) to gather the amount of CD63+ particles out of the total (Kim et al., 2019). The percentage of EXO and IDEM presenting the exosomal surface markers CD81 was determined by flow cytometry. EXO and IDEM were conjugated with Aldehyde/Sulfate Latex Beads (ThermoFisher). Briefly, 5 ml stock beads were incubated with 1 × 10^9^ EXO or IDEM for 15 min at room temperature (RT). Consequently, PBS was added to 1 ml final volume, and the samples were incubated overnight at RT under shaking. A 100 mM glycine solution was then added to saturate unbound beads and incubated for 30 min at RT before centrifugation for 5min at 4000 rpm occurred. Samples were incubated for 30 min with APC-conjugated antibody for CD81 (Biolegend). The BD LSR Fortessa^TM^ flow cytometer was employed for samples analysis. 10,000 events per sample were acquired, and the FCS/SSC parameters were used to gate nanoparticle-bound beads which have a 4 μm diameter. Data were analyzed with the FlowJo software (BD).

#### Scanning Electron Microscope

To analyze IDEM and EXO by Scanning Electron Microscope (SEM) 50 μL of each sample were placed in 60 × 15 mm petri dishes and incubated overnight at 4°C. 2.5% glutaraldehyde was subsequently added to each sample for 10 min at 4°C. Samples were then washed with ethanol at increasing percentages (30–50–70–90–100%, respectively) for 5 min each at RT. Lastly, samples were immersed in 50% butanol for 5 min. A 2 × 2 cm Petri dish piece was mounted with double side carbon tape (Ted Pella Inc., United States) on an aluminum SEM stub (Ted Pella Inc., United States). A 7 nm Iridium film was added to the sample to enhance image contrast. A Nava Nano SEM 230 (Thermal Fisher, United States) was used to image samples. All SEM experiments were performed at RT (22°C) and under a high vacuum range (5 × 10^–6^ to 2 × 10^–6^ Torr). The accelerator voltage was set at 5–7 kV for imaging. The electron beam spot-size was set at 3 nm and the working distance was 5 mm.

#### Atomic Force Microscopy

Atomic Force Microscope (AFM) Bruker BioScope Catalyst (Bruker Instruments, Santa Barbara, CA, United States) was used, and Bruker MLCT-E cantilevers were used to perform the analysis, with a nominal spring constant of 0.1 N/m. Each cantilever was calibrated for spring constant determination on a clean glass slide prior to each measurement. All imaging was done in PeakForce Tapping, with a scan rate of 1 Hz and a force of 200 pN and scans ranged from 25 to 0.5 μm^2^. Each high-resolution image contained one or few exosomes at the center of which a force curve was taken, with a ramp force of 150 pN (for elastic modulus calculation) and of 2 nN (for adhesion measurement), a ramp rate of 1 Hz and a ramp size of 150 nm. 10 ThP-1 and 25 semisynthetic exosomes were considered. Elastic modulus was obtained using the fitting modulus in the Nanoscope Analysis software v1.50 on the approach curve of each force curve and using the Hertz model, Equation 1.

$$F=\frac{4\,E\,\sqrt{R}\,\delta^{3/2}}{3\,\left(1-\upsilon^{2}\right)}$$

In Equation 1, F is the force applied, E is the Young’s modulus (fit parameter), υ is the Poisson ratio (0.5), R the radius of the indenter (20 nm), δ is the indentation depth and α is the half-angle of the indenter (18°). For adhesion evaluation, the percentage of force curves that showed an adhesion event was considered. Data was then analyzed and plotted using Mathematica 12.0. Samples were prepared as follows. Briefly, 60 μl of 0.1% of APTES solution was deposited on freshly cleaved mica flakes (1 μm^2^), left for 30 min, rinsed with 5 mL of MilliQ water and dried with N2. Then 100 μl of EXO/IDEM solution was deposited on the substrate for 2 h, mica was rinsed with 2 mL of PBS and analyzed with AFM in PBS solution.

#### Western Blot

After exosomes and IDEM preparation, proteins were extracted in 80 μL M-PER lysis buffer (ThermoFisher) and quantified against a BSA standard curve using the Pierce^TM^ BCA Protein Assay Kit (ThermoFisher). Consequently, 20 μg of proteins were run through a Mini Protean Gel (Bio-Rad) and transferred onto a PVDF membrane using a semi-wet transferring system (Bio-Rad). Membranes were then incubated for 1 h in blocking solution (BSA 5% in TBS-T solution, ThermoFisher), followed by incubation over night at 4°C in anti-CD63 rabbit primary antibody with a 1:1000 dilution (Abcam). The next day, membranes were washed in TBS-T 3 times and then incubated for 1 h at room temperature with the anti-rabbit secondary antibody in a 1:2000 dilution (ThermoFisher). Membranes were then developed using a Chemidoc XRS Molecular Imager (Bio-Rad).

### Drug Encapsulation and Encapsulation Efficiency (EE%) Assessment

Efficiently loading of doxorubicin within IDEM and EXO was achieved through saponin as an encapsulation enhancer. A 0.1% concentration of saponin was tested. Briefly, both nanoparticle solutions were mixed with 400 μg/mL of doxorubicin before adding saponin, and the mixture was incubated for 5 min at 37°C in agitation at 200 rpm. Consequently, unencapsulated doxorubicin was removed using an Exosome Spin Column (Invitrogen). After the loading, the encapsulation efficiency (EE%) was measured. To this, 0.1% of Triton-X-100 was added to the samples for 10 min at RT. The concentration of doxorubicin encapsulated in both IDEM (IDEM-DOXO) and EXO (EXO-DOXO) was determined by measuring its excitation and emission values (480 and 610 nm, respectively) against a set of known standards with a Synergy H4 Hybrid Plate Reader (Biotek).

### Drug Release Profile

To assess the release profile of the nanoparticle formulations, EXO-DOXO and IDEM-DOXO samples were injected in two Snakeskin Dialysis Membranes (10,000 MWCO) that were then immersed in a beaker with 20 mL PBS heated at 37°C. At different time points, 1 mL of PBS was taken from each beaker for measurement and replaced with 1 mL of fresh 37°C-heated PBS for up to 96 h. The doxorubicin fluorescent signal was then read with a plate reader (Biotek) and compared to a doxorubicin standard curve. The cumulative drug release was calculated according to the following equation:

$$D\,R\%=\sum_{n=1}^{n=t}\frac{\left(C_{n}\cdot {\text{V–C}}_{n-1}\cdot v\right)}{m_{0}}\cdot 100$$

Where *t* is the number of time points, *C*_*n*_ is the concentration of doxorubicin at time t, *V* the total volume of liquid (in our case 20 mL), *C*_*n–1*_concentration of doxorubicin at time t-1, *v* non-withdrawn volume (in our case 19 mL), *m*_*0*_ the mass of doxorubicin in the nanoparticles.

### IDEM- and EXO-DOXO Uptake by Ovarian Cancer Cells

#### Confocal Microscopy

To assess the IDEM- and EXO-mediated uptake of doxorubicin by ovarian cancer cells, 1 × 10^4^ SKOV3 cells were seeded in 8-well of chamber glass slide and cultured overnight. The next day, 1 and 3 μg/mL doses of EXO-DOXO and IDEM-DOXO were added and left for up to 12 h. At 3 different time points (2, 4, 12 h) cells were washed twice in pre-warmed PBS at pH 7.4, fixed in 4% paraformaldehyde for 10 min at RT and permeabilized in 0.1% Triton X-100 in PBS for 5 min. After washing in PBS twice, cells were stained with phalloidin Alexa-Fluor 488, diluted 1:200 in PBS, and incubated for 20 min at RT before staining with DAPI Prolong anti-fade mountant (Invitrogen) occurred. Samples were imaged with Fluoview TM3000 confocal microscope (Olympus).

#### Flow Cytometry

In order to quantify the percentage of cells containing doxorubicin, 1 × 10^5^ SKOV-3 cells were seeded in 12 multi-well plates and cultured overnight. The next day, 1 and 3 μg/mL doses of EXO-DOXO and IDEM-DOXO were added to the cells and incubated for 12 h. A free DOXO formulation was added as a positive control at a 10 μg/mL concentration. At three different time points (2, 4, 12 h) cells were detached and analyzed for the doxorubicin signal expression at the excitation of 480 nm and emission at 590 nm (corresponding to the PerCP-Cy5.5 channel). 10,000 events per sample were acquired with a BD LSR Fortessa flow cytometer, and the FCS/SSC parameters were used to gate cells.

### Cytotoxic Effect of IDEM- and EXO-DOXO in 2D

SKOV-3 cells viability in 2D conditions after treatment with EXO-DOXO and IDEM-DOXO was assessed *in vitro* using 2 complementary techniques, the Alamar blue assay and the impedance-based cell index measures, respectively.

#### Alamar Blue Assay

SKOV-3 cells were seeded at the density of 6000/well on a 96-well plate and incubated overnight. The following day they were treated with different concentrations of doxorubicin encapsulated EXO and IDEM (namely, 1, 3, and 5 μg/mL). Cell viability was assessed for up to 96 h from the quantification of the resazurin dye reduction (Ansar Ahmed et al., 1994). Cytotoxicity was measured by growth inhibition and was plotted as a surviving percentage over time. At each time point (24, 48, 72, 96 h, respectively) the absorbance signal of the cells was read at 570 nm and normalized to the values read at 600 nm with a plate reader (Biotek). Three biological replicates were performed and the growth of nanoparticle-treated cells was compared to untreated controls. Free doxorubicin (at the concentration of 10 μg/mL) was used as a positive control.

#### eSight

The xCELLigence RTCA eSight machine (Agilent) was used to obtain real-time impedance-based measurements of cell adhesion and proliferation (Chollangi et al., 2018). The user can set a temporal resolution (how frequent the impedance value is measured) in which an adimensional value called “cell index” is measured. SKOV-3 cells were seeded at the density of 6000/well in a company-provided electronic 96-well microplate and cultured overnight. The following day, the same doses of nanoparticles used for the Alamar blue assay were added, and the cell index was measured for up to 96 h. Data were analyzed with the CELLigence RTCA Software and plotted as cell index values over time. Right before adding the treatments, cells in each well were also stained with 2 μM of the fluorescently labeled Caspase-3 dye (ACEA Biosciences) which is a marker for early apoptotic events. Four images per well were taken by the eSight machine at a 10X magnification every hour using the DAPI channel (429/469 nm), and all signal-positive cells were counted and plotted by the RTCA Software as object counts/well over time.

### Cytotoxic Effect of IDEM- and EXO-DOXO in 3D Culture Systems

Spheroids were produced using Ultra Low Attachment (ULA) plates (Corning). Briefly, SKOV-3 cells were seeded at the density of 5000 cells/well in a Corning ULA 96-well plate and cultured overnight. After 24 h, EXO-DOXO and IDEM-DOXO were added to achieve the concentration of 5 μg/mL of doxorubicin encapsulated. The cell viability of the spheroids was assessed using the CellTiter-Glo 3D reagent assay (Promega) at different time points (24, 48, 72, 96 h respectively) for up to 96 h. Briefly, the reagent was heated at 22°C prior to use and added to experimental wells for 30 min at RT in 1:1 volume ratio. The plate shaking was essential for a proper spheroids lysis. After the incubation time, the luminescent signal was read with a plate reader (Biotek). Free doxorubicin (at the concentration of 10 μg/mL) was used as a positive control. For each time point, a representative 10X picture of each spheroid was taken with a IncuCyte Live-Cell analysis system (Essen Bioscience).

### Statistical Analysis

For all statistical analysis including size, concentration, encapsulation efficiency and drug release, cell cytotoxicity in both 2D and 3D experiments, a two-tailed Student’s *t*-test was performed to compare IDEM and EXO. Data with *p* < 0.05 were considered significant (^∗^*p* < 0.05, ^∗∗^*p* < 0.01, ^∗∗∗^*p* < 0.001). All graphs show average values and standard deviation.

## Results

### EXO and IDEM Synthesis and Characterization

**FIGURE 1:**
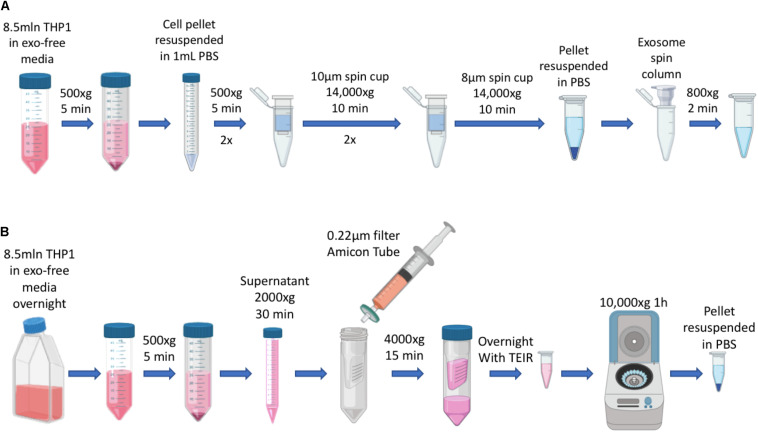
Schematics of EXO and IDEM production. **(A)** Summary of IDEM production through filtered-membrane centrifugation steps. **(B)** Summary of EXO extraction from culture media by using the Total Exosome Isolation Reagent (TEIR).

Exosomes (EXO) and IDEM were obtained as summarized in Figure 1.

**FIGURE 2:**
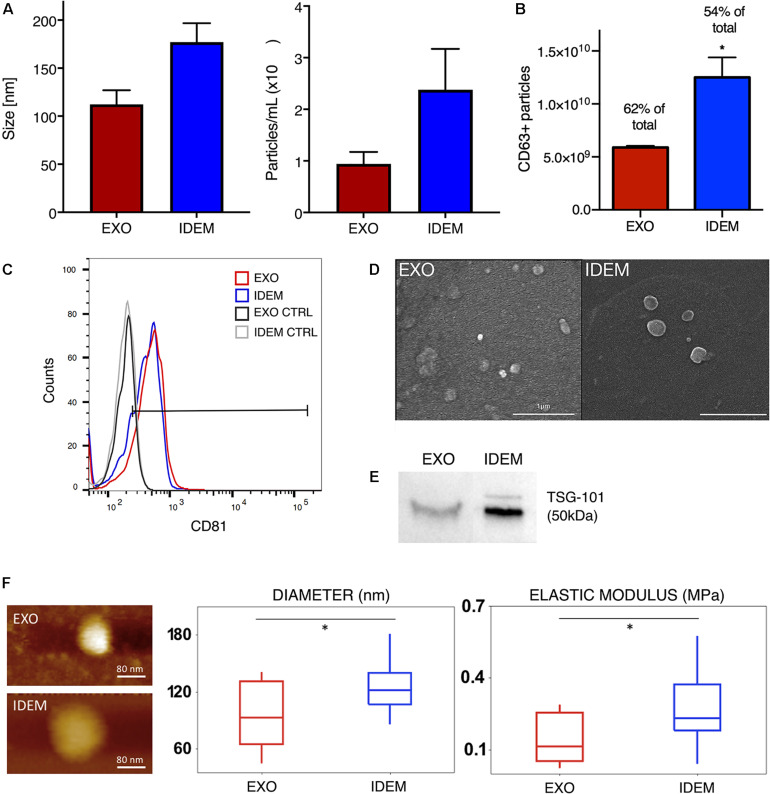
EXO and IDEM characterization panel. **(A)** Size (nm) and concentration (particle/ml) values for EXO (in red) and IDEM (in blue) obtained by NTA (*n* = 11). Statistically significant differences (***p < 0.001) were observed between the two particle types formulations. In particular IDEM were 2.48 times more concentrated than exosomes. **(B)** Number of CD63-positive EXO and IDEM as measured by ELISA (*p < 0.05). **(C)** Flow cytometric analysis showing EXO and IDEM stained with a APC-CD81 antibody. Results show the positive signal compared to unstained exosomes. **(D)** Scanning electron microscope (SEM) images show rounded particles phenotypes. **(E)** Western blot analysis of TSG-101 shows the exosomal marker presence on both EXO and IDEM. **(F)** Topography and Elastic Modulus of EXO and IDEM. (Left) A representative AFM image of each formulation. (Middle) Boxplot data for diameters size for EXO and IDEM. Mean EXO value: 96 ± 35 nm. Mean IDEM value: 129 ± 34 nm. (Right) Boxplot data for Elastic Modulus for EXO and IDEM. Mean EXO value: 0.15 ± 0.10 MPa. Mean IDEM value: 0.28 ± 0.14 MPa (*p < 0.05).

Nanoparticle tracking analysis (NTA) was used to determine size and concentration of the two formulations. As shown in Figure 2A, EXO and IDEM presented an average size of 112 ± 14 nm and 177 ± 19 nm, respectively (*p* < 0.001). Starting from the same number of ThP-1cells (8.5 × 10^6^), the optimized procedure allowed for the production of 2.3 × 10^10^ IDEM compared to 9.4 × 10^9^ EXO obtained following standard protocols for the isolation of natural exosomes from culture media. The difference in particle concentration was statistically significant between the two nanoparticle types, IDEM yielding a 2.48 times higher concentration than EXO (*p* < 0.001). The presence of exosomal markers, the percentage and number of particles expressing CD63 and CD81 were confirmed by flow cytometry and ELISA, respectively. Figure 2B shows the relative number of CD63-positive EXO and IDEM compared to total particles obtained by NTA analysis. The number of CD63-positive EXO obtained by ELISA was 5.89 × 10^9^ (which represents the 62% of the total number) while 1.25 × 10^10^ of IDEM were CD63-positive (54%). The presence of the CD81 surface exosomal marker was also confirmed for both particle types by flow cytometry; 43.5% for IDEM and 40.9% for EXO (Figure 2C). Western blot analysis was also performed to assess the presence of the TSG-101 marker, a cytosolic protein highly expressed in exosomes, and confirmed its presence on both EXO and IDEM (Figure 2E). Characterization data were corroborated by scanning electron microscopy (Figure 2D) and AFM images (Figure 2F) that provided morphological information about the two nanoparticle types. AFM was used to gather insights into the surface, stiffness and adhesion properties of the nanoparticles. Tapping mode analysis showed a general deviation from the globular, near spherical shape expected for exosomes, while IDEM presented a more uniform, globular shape. The mean radius was significantly smaller, 95.8 ± 35 nm compared to 129.4 ± 34 nm for EXO and IDEM, respectively (*p* < 0.05). EXO had a significantly lower elastic modulus of 0.15 ± 0.1 MPa compared to IDEM, 0.28 ± 0.14 MPa (*p* < 0.005). The adhesive properties of the two nanoparticles were similar, with non-specific tip-particle adhesion events recorded as 36% for EXO and 38% for IDEM.

### Encapsulation and Drug Release Profile

**FIGURE 3:**
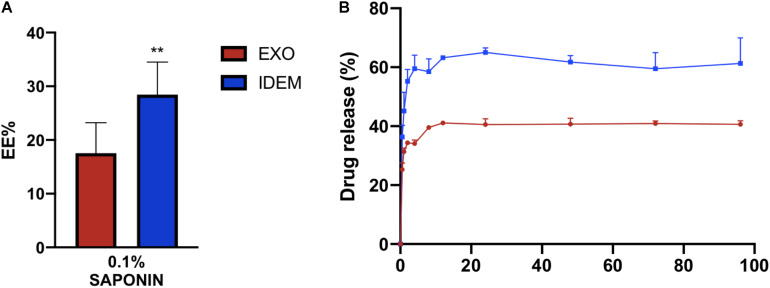
Encapsulation efficiency (EE%) and cumulative drug release profile. **(A)** EE% values obtained from a doxorubicin starting concentration of 400 μg/mL. IDEM show higher EE% than EXO (**p < 0.01). **(B)** Drug release profile (%) of doxorubicin from IDEM and EXO over a 96-h period. A burst release was seen for both formulations for up to 12 h, followed by a plateau reached soon after.

EXO and IDEM were loaded with doxorubicin using 0.1% saponin. Figure 3A shows the encapsulation efficiency (EE%) values normalized to the initial doxorubicin concentration (400 μg/mL). IDEM showed 28% encapsulation efficiency while EXO retained 17% of the initial administered drug. After assessing drug encapsulation, the cumulative drug release from both EXO and IDEM was determined. Figure 3B shows the percentage of drug released over a 96-h window. Both nanoparticle formulations showed a pronounced burst release that reached a plateau after about 12 h. IDEM proved to release 60% of drug after 12 h, whereas EXO 40% of the encapsulated amount.

### Nanoparticles Effect on the Proliferation and Early Apoptosis of Ovarian Cancer Cells

**FIGURE 4:**
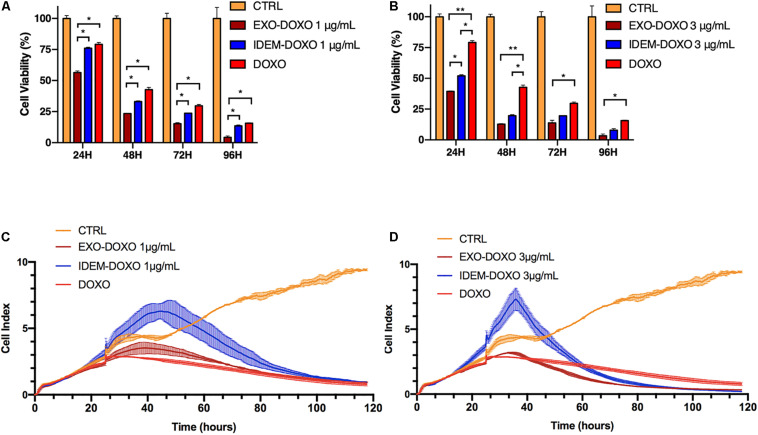
Cell proliferation assessed by Alamar blue and xCELLigence eSight analysis of cell index. **(A)** Percentage of viable cells following the treatment with doxorubicin-loaded IDEM (IDEM-DOXO) and EXO (EXO-DOXO) at 1 μg/mL. Alamar blue assay was used to quantify the percentage of viable cells over a 96-h period. IDEM- and EXO-DOXO are statistically more cytotoxic than the free doxorubicin formulation (10 μg/mL, DOXO) at 48, 72, and 96 h time points (p* < 0.05). A two-tail *T*-test was performed to assess for statistical significance compared to free doxorubicin formulation (DOXO, p* < 0.05). **(B)** Percentage of SKOV-3 viable cells following the treatment with IDEM-DOXO and EXO-DOXO at 3 μg/mL. EXO-DOXO were more effective than free doxorubicin (DOXO) in causing cell cytotoxicity on SKOV-3 cells at all time points (p** < 0.01 at 24 and 48 h, p* < 0.05 at 72 and 96 h respectively) while IDEM-DOXO were significantly more effective at 24 and 48 h time points (p* < 0.05), both formulations reaching more than 50% reduction in viability as soon as 24 h after the beginning of the experiment. **(C)** Cell index analysis of 1 μg/mL of IDEM-DOXO and EXO-DOXO over time, showing a marked decrease in cell index compared to untreated control after up to 110 h. **(D)** Cell index analysis of 3 μg/mL of IDEM-DOXO and EXO-DOXO have a similar effect to free DOXO in reducing cell proliferation of SKOV-3 cells for a time period of up to 110 h.

Cytotoxic effects on ovarian cancer cells (SKOV-3) mediated by IDEM and EXO loaded with doxorubicin was evaluated using Alamar blue. Three increasing concentrations (1, 3, and 5 μg/mL) of doxo-encapsulated IDEM (IDEM-DOXO) and EXO (EXO-DOXO) were added to SKOV-3 cell culture media. Free doxorubicin (DOXO, 10 μg/mL) was used as a positive control. This dose was identified as the lowest and most cytotoxic among different concentrations of doxorubicin tested on cell viability (data now shown). Figure 4A shows a significant effect of IDEM-DOXO on SKOV-3 cells, with a 25% reduction in cell viability at 24 h following the treatment at the lowest treatment dose (1 μg/mL). Viability decreased further to 70, 75, and 80% at 48, 72, and 96 h respectively. EXO-DOXO at a 1 μg/mL concentration showed a statistically more enhanced effect compared to IDEM, again being more cytotoxic than free DOXO at 24 h (*p* < 0.05). Increasing the dose of IDEM to 3 μg/mL resulted in an even more pronounced cytotoxic effect than free DOXO (Figure 4B), with a 50% reduction in cell viability occurring at 24 h (*p* < 0.05). This cytotoxic effect further increased to 85% reduction in cell viability after 72 h. In comparison, the EXO-DOXO treatment brought to a reduction in viability in a faster fashion, reaching more than 90% cell death at 96 h (*p* < 0.05).

Treatments with empty nanoparticles confirmed the cytotoxic effect was mediated by the encapsulated doxorubicin, and exclude an inherent nanoparticle effect (data shown in Supplementary Figure S1A).

To substantiate the Alamar blue assay data, the efficacy of IDEM-DOXO on cell adhesion and proliferation was also tested by measuring impedance changes due to cell death resulting in cell detachment from culture plates (xCELLigence eSight). Figure 4C shows the effect of 1 μg/mL of IDEM-DOXO on the cell index of SKOV-3 cells, which represents a non-dimensional value directly correlated to cell proliferation. The IDEM-DOXO treatment showed a peak in cell index (=6) in the 40–50 h time window, followed by a marked reduction in line with the cell index values of free DOXO and EXO-DOXO. After 110 h, the cell index of all three treatments was less than 1, indicating an almost complete decrease in cell viability. A similar trend was observed when IDEM-DOXO were tested at a 3 μg/mL concentration (Figure 4D). The cell index reached a peak value of 7 in the 35–45 h time window, followed by its decrease in value to 0.26 after 110 h. Free DOXO and EXO-DOXO reached a cell index value 0.87 and 0.35 after 110 h, respectively. Again, nanoparticles confirmed that the reduction in proliferation was caused by the encapsulated doxorubicin only (data shown in Supplementary Figure S1B).

**FIGURE 5:**
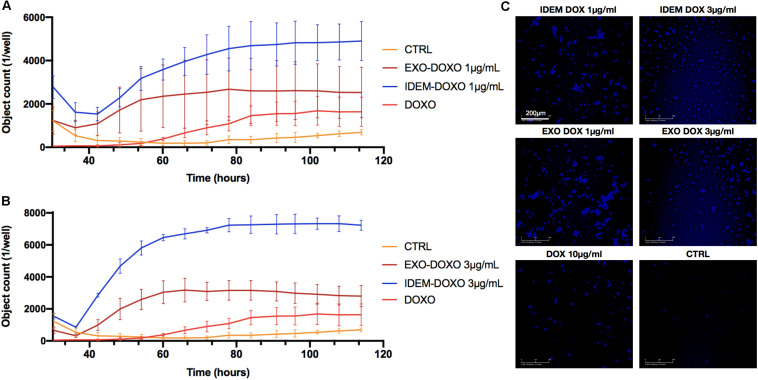
Quantification of early apoptosis. **(A)** Analysis of Caspase3 positive cells after treatment with 1 μg/mL of IDEM-DOXO and EXO-DOXO over a 110-h time period. Ten μg/mL of free doxorubicin (DOXO) was used as a positive control. Pictures were taken every hour and analyzed with the RTCA Software. **(B)** Analysis of caspase3 positive cells after treatment with 3 μg/mL of IDEM-DOXO and EXO-DOXO, with IDEM being the most effective in causing early apoptotic events. **(C)** Representative images that show the signal intensity which is directly correlated to early apoptotic events. It can be noticed how all treatments lead to marked early apoptotic events when compared to the untreated control. A 10X magnification objective was used for pictures, with a scale bar of 200 μm.

Apoptosis was also assessed following IDEM-DOXO and EXO-DOXO treatment using the eSight system by combining data for expression levels of the apoptotic marker Caspase3 with data extrapolated from the proliferation assay, and correlating the number of Caspase3 positive cells with pro-apoptotic activity. 1 and 3 μg/mL treatments with IDEM-DOXO yielded 4900 ± 900 and 7230 ± 314 caspase positive cells after 110 h, respectively, which was higher than the free DOXO and EXO-DOXO treatments (Figures 5A,B). For EXO-DOXO treatment 2520 ± 1160 and 2790 ± 660 positive cells were identified after 1 and 3 μg/mL treatments, while free DOXO yielded 1630 ± 670 positive cells. A clear increase in the number of Caspase-3 positive cells can be seen when comparing both the untreated and the free DOXO-treated cells with the exosomes and IDEM treatments (Figure 5C). No apoptotic effect was seen when empty nanoparticles were tested as controls (data shown in Supplementary Figure S2).

### Doxorubicin Uptake

**FIGURE 6:**
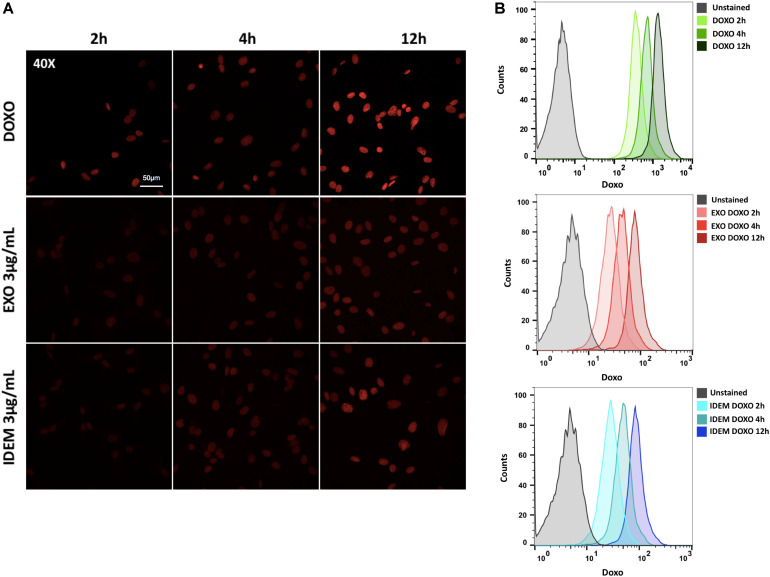
Doxorubicin uptake analysis. **(A)** Confocal microscopy images showing an increasing signal associated to free doxorubicin (DOXO, 10 μg/ml), EXO-DOXO (3 μg/ml), and IDEM-DOXO (3 μg/ml) uptake by SKOV-3 cells overtime (2, 4, 12 h). 40x Magnification, with a 50 μm scale bar. **(B)** Flow cytometry was used to quantify the amount of doxorubicin encapsulated at 2, 4, 12 h following cell treatment. The PerCP-Cy5.5 channel was used to detect doxorubicin autofluorescence signal and was compared to untreated cells to set the unstained signal. Both IDEM-DOXO and EXO-DOXO show increased signal intensity over time, however, free doxorubicin displays the brightest signal following uptake by SKOV-3 cells.

A 3 μg/mL dose of both exosomes and IDEM was added to SKOV-3 cells for 2, 4, and 12 h to evaluate DOXO uptake. The autofluorescent signal intensity of DOXO increasing over time was inferred from microscopy images (Figure 6A) and quantified using flow cytometry. Both, IDEM-DOXO and EXO-DOXO show a similar pattern of incremental uptake by SKOV-3 cells with slightly less uptake than free doxorubicin (Figure 6B).

### Cytotoxic Effect of IDEM on Ovarian Cancer Cells in 3D

**FIGURE 7:**
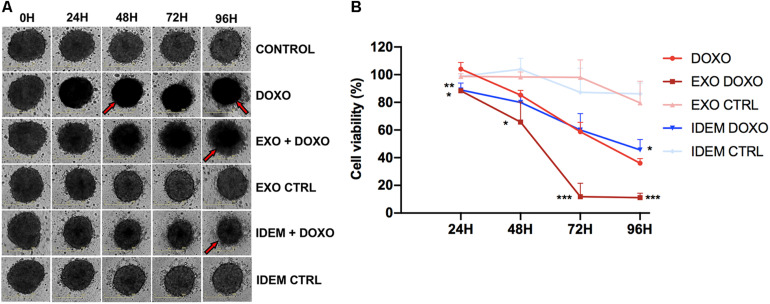
Effect of EXO and IDEM on 3D ovarian cancer model. **(A)** Representative images taken at 24, 48, 72, and 96 h following the treatment with 5 μg/mL of IDEM-DOXO and EXO-DOXO and 10 μg/mL of free doxorubicin (DOXO), while empty EXO and IDEM were used as negative controls (IDEM CTRL and EXO CTRL, respectively). Untreated cells are included as negative control (CTRL). The effect of doxorubicin is evident in increasing necrosis and reducing spheroids integrity, as indicated by the red arrows. **(B)** Percentage of cell viability normalized to the untreated control of all treatments obtained with the CellTiter Glo viability assay. IDEM-DOXO were significantly more effective than DOXO at 24 and 96 h time points (*p < 0.05) while EXO-DOXO were the most effective at all time points (***p < 0.001 at 72 and 96 h).

After testing the effect of the nanoparticle treatments in 2D systems, we evaluated their effect in a more complex spheroid system which better mimics the *in vivo* environment. 24 h post-spheroid formation, cells were treated with 5 μg/mL of IDEM-DOXO and EXO-DOXO and monitored for up to 96 h. The presence of doxorubicin, both as a free formulation and encapsulated in IDEM or EXO, affects the shape and the color of spheroids causing them to lose integrity and to become darker, as can be noticed by eye in Figure 7A. These phenotypical changes might be due to increased levels of necrosis in the spheroids inner core, as has been extensively reported in literature (Freyer, 1988; Sokolova et al., 2019). These effects were corroborated by assessing cell viability; IDEM-DOXO particles were as effective as EXO-DOXO, and both formulations were more cytotoxic than free doxorubicin in reducing spheroids proliferation by almost 20% after 24 h (*p* < 0.01 and <0.05, respectively; Figure 7B). IDEM-DOXO showed the same effect as doxorubicin at 48 and 72 h, but its effects were significantly greater after 96 h (*p* < 0.05). Similarly, doxorubicin-loaded exosomes were significantly more effective than DOXO at 72 and 96 h (*p* < 0.001).

## Discussion

Due to their role in intercellular communication, exosomes are being increasingly explored as delivery systems for biomedical purposes. In particular, the organotropism of exosomes toward tumor sites and metastatic niche makes them ideal candidates for the development of efficient anticancer therapies targeting metastatic cancer with reduced side effects. However, the main drawbacks for the application of naturally occurring exosomes are the challenges of large-scale production, isolation, modification and purification (Ha et al., 2016; Akuma et al., 2019). Exosome mimetics hold the promise to improve the delivery of bioactive molecules with therapeutic efficacy, while achieving scalability and increasing bioavailability due to their minimal immunogenicity (Antimisiaris et al., 2018). In this work we propose a system to scale up the production of exosome mimetics from immune cells. By subjecting cells to serial extrusion steps through filters with diminishing pore sizes and by consequently loading them with a chemotherapeutic agent, we generated high quantities of exosome-mimetic nanovesicles carrying sheltered drugs. Compared to standard procedures for the production of exosomes, our approach allows for a rapid synthesis and purification, which represents a practical advantage in terms of production.

Starting from the same number of cells (8.5 million ThP-1 cells), our approach led to a 2.5-fold increase in the number of IDEM particles obtained, compared to naturally released exosomes (EXO). Despite the greater average diameter of IDEM compared to EXO (177 ± 19 nm vs. 112 ± 14 nm, respectively), IDEM remain within the size-window that is expected for exosomes (Doyle and Wang, 2019), and retain the typical molecular and mechanical features of natural exosomes. IDEM tested positive for the tetraspanins CD81 and CD63, the presence of which has been widely associated with endosomal-derived exosomes (CD81) (Greening et al., 2017; Hikita et al., 2018) and have been proved to be cell-type dependent (CD63) (Kowal et al., 2016). Levels of CD81 and CD63 found in IDEM were comparable to those found in exosomes isolated from the media of the same cell population, confirming their similarity with their physiological counterparts.

Through SEM and AFM analysis, three-dimensional morphological images of both particles were generated showing a spheroidal shape. Size measurements performed by AFM for both particle types followed the same trend obtained by NTA, although average particle size was lower when measured with AFM, and attributed to the different measurement techniques (Sharma et al., 2010, 2011; Paolini et al., 2016). Nanomechanical analysis revealed that both particle types had same adhesive characteristics indicating similar surface composition (Whitehead et al., 2015).

In contrast, IDEM particles had a greater elastic modulus indicating an increased stability. A greater elastic modulus has been as well associated with a better cellular uptake efficiency (Sun et al., 2015; Wang et al., 2019), although this effect has not been observed in the present study.

IDEM proved to be an effective drug delivery system when loaded with the chemotherapeutic agent doxorubicin which is currently the standard of care approach for the treatment of metastatic ovarian cancer (Gordon, 2005; Cronin et al., 2013). Efficient doxorubicin encapsulation was achieved using saponin treatment yielding 28 and 17% drug encapsulation for IDEM compared to EXO respectively. This further demonstrates the enhanced benefits of IDEM over EXO, since the encapsulation efficiency of doxorubicin does not generally exceed 20% in exosomes (Gomari et al., 2018; Wei et al., 2019). Moreover, IDEM released an amount of drug over time up to 20% greater than exosomes.

Exosomes deploy different characteristics that render them an attracting system for cancer therapy. For instance, they can be taken up by acceptor cells (and alter cellular processes), and can avoid blood clearance by the immune system (Antimisiaris et al., 2018). These aspects together with the nanoscopic size are likely to permit their penetration into tumor bulks or in reaching secondary metastatic sites, which represent one of the main challenges in ovarian cancer treatment specifically (Mitra, 2016; Cheng et al., 2017). IDEMs encompass these exosomes features and provide a suitable alternative as they are reconfigurable and more clinically translatable due to their scalability properties. In order to assess the applicability of IDEM in an ovarian cancer setting SKOV-3 cells were used to evaluate the cytotoxic effect of doxorubicin-loaded IDEM. Interestingly, both IDEM and EXO proved to be more efficient than the free drug despite DOXO being delivered at a lower drug concentration, with the EXO-DOXO treatment being statistically more effective than IDEM-DOXO as well. This event might be due to different uptake mechanisms of EXO-DOXO/IDEM-DOXO by SKOV-3 cells compared to the free Doxorubicin, leading to a more enhanced cytotoxic effect of the chemotherapeutic agent. Also, as highlighted from the higher cytotoxic effect of EXO CTRL on SKOV-3 cells compared to IDEM, we speculate additional exosomal components might be involved in the effect mediated by EXO which are not present in IDEM. Moreover, drug efflux pumps which expel drugs from cells are known to be at the base of multi-drug resistance (Filipits, 2004). In contrast, liposomal-based nanoformulations, which have a similar theoretical composition to EXO and IDEM, have been proven to increase retention of drug into cells, thus increasing their efficacy (Patel et al., 2013).

In addition to these findings, IDEM-DOXO effectively reduced cell proliferation at the concentrations of 1 and 3 μg/ml, where the cell index analysis revealed two peaks in the 38–45 h window. This might be explained with an initial cell bloating, corresponding to increased impedance levels, followed by cell disruption resulting in cell index decrease. Doxorubicin-loaded IDEM also induce the highest rates of caspase3-dependent early apoptotic events in SKOV-3 cells compared to EXO, allowing us to evaluate the extent and timing of this mechanism. In the case of our IDEM, this could be an indication of a better efficacy index. Moreover, the higher apoptosis ratios after the IDEM-DOXO treatment might be explained with different uptake rates or possibly differential uptake mechanisms by SKOV-3 cells, which might lead to a faster activation of the apoptotic cascade.

In order to gain more information about the behavior of our platform in more complex systems, the cytotoxic effect of doxorubicin-loaded IDEM was tested on SKOV-3-generated spheroids. Data obtained show again a more enhanced activity when compared to the free doxorubicin formulation. Although the efficacy of doxorubicin in penetrating spheroid systems resembling tumor masses to exert its effect has been widely established (Sokolova et al., 2019), our results support the idea that the addition of exosome-like delivery systems can further improve the chemotherapeutic penetration and, hence, the efficacy of the therapy.

Overall, we demonstrate the feasibility of our IDEM platform as an effective alternative to exosomes for the development of semi-synthetic nanoparticles for ovarian cancer therapeutics. Compared to EXO, the advantages IDEM offer include the rapid synthesis and purification, the higher production yields, and an increased encapsulation efficiency/release, which make them potential candidates for clinical translation. Moreover, the use of the ThP-1 monocytic cell line for IDEM production not only has a practical advantage due to its ease of expansion in laboratory culture systems, but also proves convenient for the development of anti-cancer strategies with a potential application in the more complex environment found in *in vivo* settings, where the immune system plays a crucial role. ThP-1 cells can help investigate the interaction of cancer systems with different macrophages types (these cells can also be differentiated in M0/M1/M2 macrophages), allowing us to further deepen our understanding of the potential of IDEM derived from different macrophage types to efficiently interact with OC systems.

Exosomes retain parent cell characteristics in terms of surface markers, content and cell pathway activation, properties that assume a pivotal role when related to immune cells, particularly in terms of tumor recognition to exert an effective response (Wen et al., 2017). Notably, exosomes derived from precursor monocyte populations do not exhibit the pro-active inflammatory properties associated with their “offspring” macrophages highlighting the importance that the source of exosomes play in activating or suppressing the immune response following administration (Rice et al., 2018). As such, the use of monocyte-derived exosome mimetics combines the advantages of a biomimetic drug-delivery system with reduced immunogenicity as they should not induce systemic inflammation following infusion nor face being rejected by the immune system. Regardless of the lower *in vitro* efficacy of IDEM-DOXO when compared to EXO-DOXO, our exosome mimetics still outperformed the free drug treatment. Hence, we believe IDEM will contribute to revolutionizing the treatment of malignancies for which a semi-synthetic, highly efficient, reproducible, and fast approach is paramount.

## Data Availability Statement

The raw data supporting the conclusions of this article will be made available by the authors, without undue reservation.

## Author Contributions

SP and BC contributed to the conception and design of the study. SP, IP, AG, JG, and LWF contributed in the acquisition and analysis of data for the work. SP, BC, AG, LWF, DG, and RSC contributed in the interpretation of data for the work. SP and BC wrote the first draft of the manuscript. SP, BC, and RSC contributed to the manuscript revision, read, and approved the submitted version. All authors contributed to the article and approved the submitted version.

## Conflict of Interest

The authors declare that the research was conducted in the absence of any commercial or financial relationships that could be construed as a potential conflict of interest.
